# The effects of vitamin D on all-cause mortality in different diseases: an evidence-map and umbrella review of 116 randomized controlled trials

**DOI:** 10.3389/fnut.2023.1132528

**Published:** 2023-06-22

**Authors:** Mingyu Cao, Chunrong He, Matthew Gong, Song Wu, Jinshen He

**Affiliations:** ^1^Department of Orthopaedic Surgery, Third Xiangya Hospital of Central South University, Changsha, China; ^2^Department of Orthopaedic Surgery, University of Pittsburgh, Pittsburgh, PA, United States

**Keywords:** vitamin D, all-cause mortality, umbrella review, patients, COVID-19

## Abstract

**Purpose:**

To conduct a solid evidence by synthesizing meta-analyses and updated RCTs about the effects of vitamin D on all-cause mortality in different health conditions.

**Methods:**

Data sources: Pubmed, Embase, Web of Science, the Cochrane Library, Google Scholar from inception until 25th April, 2022. Study selection: English-language, meta-analyses and updated RCTs assessing the relationships between vitamin D and all-cause mortality. Data synthesis: Information of study characteristics, mortality, supplementation were extracted, estimating with fixed-effects model. A Measurement Tool to Assess Systematic Reviews, Grading of Recommendations Assessment, Development and Evaluation, and funnel plot was used to assess risk of bias. Main outcomes: All-cause mortality, cancer mortality, cardiovascular disease mortality.

**Results:**

In total of 27 meta-analyses and 19 updated RCTs were selected, with a total of 116 RCTs and 149, 865 participants. Evidence confirms that vitamin D reduces respiratory cancer mortality (RR, 0.56 [95%CI, 0.33 to 0.96]). All-cause mortality is decreased in patients with COVID-19 (RR, 0.54[95%CI, 0.33 to 0.88]) and liver diseases (RR, 0.64 [95%CI, 0.50 to 0.81]), especially in liver cirrhosis (RR, 0.63 [95%CI, 0.50 to 0.81]). As for other health conditions, such as the general health, chronic kidney disease, critical illness, cardiovascular diseases, musculoskeletal diseases, sepsis, type 2 diabetes, no significant association was found between vitamin D and all-cause mortality.

**Conclusions:**

Vitamin D may reduce respiratory cancer mortality in respiratory cancer patients and all-cause mortality in COVID-19 and liver disorders' patients. No benefits showed in all-cause mortality after vitamin D intervention among other health conditions. The hypothesis of reduced mortality with vitamin D still requires exploration.

**Systematic review registration:**

https://www.crd.york.ac.uk/PROSPERO/display_record.php?RecordID=252921, identifier: CRD42021252921.

## 1. Introduction

The relationship between vitamin D and mortality has become a spotlight topic of increasing research interest, evidenced by the ever-expanding body of scientific literature on the topic at present (1). This growing interest is likely due to the high prevalence of vitamin D deficiency in patients with numerous life-threatening diseases, such as COVID-19 and cancer (2), which may greatly affect the mortality rate. As a result, vitamin D supplementation has emerged as a potential treatment to reduce mortality. However, blindly taking high-doses of vitamin D without any scientific guidance has become an universal phenomenon in popular health. For example, in the U.S., the average daily intake of vitamin D has reached 100 mcg (4, 000 IU) or more for an adult (3). This pattern of use is not only causing increased healthcare costs, but having an unknown impact on its clinical effectiveness or even all-cause mortality (4). Numerous researches have been carried out to uncover the exact relationship between vitamin D and all-cause mortality in order to guide the scientific use of vitamin D.

In 1999, Bostick et al. conducted a research about the relationship between vitamin D intake and ischemic heart disease mortality, and presented a non-significant result (5). More recently, Zittermann et al. pointed out that vitamin D deficiency is associated with excess mortality explicitly, and advocated for the urgent need to clarify the correlation between vitamin D and survival in specific patient populations in 2009 (6). Since then, several landmark randomized controlled trials (RCTs) and meta-analyses assessing the effect of vitamin D on mortality have been published. However at the same time, it was from this growing body of literature that much controversy over the utility of vitamin D began to emerge. The first notable research controversy arose in 2014, where among countless studies, a conclusion that vitamin D may reduce all-cause mortality was clearly proposed by Bjelakovic et al. (7) and Bolland et al. (8). This result was widely accepted at first, but was vehemently questioned by studies published in 2019. Zhang et al. definitively suggested that no statistically significant difference was observed between vitamin D and placebo groups in both all-cause mortality and cardiovascular diseases (CVD)-related mortality. However, there did exist a significant discrepancy between these two groups for cancer-related mortality (9). Surprisingly, these conclusions regarding the effect of vitamin D on cancer-related mortality were overturned again with a non-significant conclusion in 2022 (10). To date, these fierce debates are still ongoing and unsettled. Due to the jagged quality of the evidence, the controversy related to this topic has been difficult to clarify.

Within this context, an umbrella review of prior meta-analyses and systematic reviews of RCTs may fill this gap and obliterate some of the controversy in previously published studies. An umbrella review is a popular method for systematically assessing evidence from multiple sources and delivering the highest level of evidence, due to the minimizing bias and outstanding breadth and validity of such a study design (2, 11, 12). Whether vitamin D can reduce all-cause mortality is unclear. Thus, we sought to conduct a comprehensive umbrella review of existing meta-analyses of RCTs, in order to generate an evidence map for the effects of vitamin D on all-cause mortality in different populations. It was hypothesized that vitamin D intake may only reduce mortality in populations with specific health conditions.

## 2. Methods

### 2.1. Protocol, registration, and study design

An umbrella review was conducted to estimate the effects of vitamin D on all-cause mortality in populations with different health conditions, with a comprehensive evidence collection and critical evaluation performed on the existing body of literature of meta-analyses exploring this topic (13). This review was performed following the Preferred Reporting Items for Systematic Reviews and Meta-Analyses (PRISMA) guidelines (14). The pre-specified protocol was registered 3rd June 2021 to PROSPERO prior to conducting this review (https://www.crd.york.ac.uk/PROSPERO/display_record.php?RecordID=252921; Registration number: CRD42021252921).

### 2.2. Data sources and search strategy

Pubmed, Embase, Web of Science, the Cochrane Library, Google Scholar were queried using the following search terms: “Vitamin D” + “Mortality” + Patient” + “Meta-analy^*^” OR “Metaanaly^*^” OR “Systematic review.” From these search results, we extracted meta-analyses published in English involving human patients exploring the relationship between vitamin D and mortality from inception through 25^th^ April 2022. In addition, eligible RCTs were also screened. We excluded the RCTs already involved in the existed meta-analyses, rest of which were identified as additional updated RCTs from the search beginning until 25^th^ April 2022. Additional sources included bibliographies of correlative references and studies.

### 2.3. Study selection and eligibility criteria

The predetermined eligibility criteria were systematic reviews, meta-analyses of RCTs and updated RCTs in recency assessing the efficacy of vitamin D on mortality or death outcomes of interest in patients with different diseases, which were all written in English. We included the above three types of studies regardless of the form, dosage, intaking methods of vitamin D, the baseline characteristics (clinical setting, age, sex, or race) of the examined population and the date of publication.

More specifically, systematic reviews and meta-analyses of observational cohort studies, narrative reviews or those reporting effects of vitamin D on other outcomes [e.g., vitamin D receptor (VDR), vitamin D metabolism gene polymorphisms or multiple interventions] were excluded. Letters, editorials, and articles published in languages other than English were also excluded. Composite systematic reviews or meta-analyses of RCTs and observational studies were reviewed only for data related to RCTs. For some systematic reviews and meta-analyses with overlapping or redundant interventions and outcomes; the most recent, largest, and updated study was included, unless there were concerns with quality of the study's design. Existing umbrella reviews were excluded, but were reviewed for any systematic reviews or meta-analyses not captured in the initial literature search.

Systematic reviews, meta-analyses and recent RCTs catering to pre-defined search strategies and inclusion criteria were conducted by two authors (MC, CH) under the supervision of another author (JH). Study selection was performed in a four-stage process. Firstly, duplications were removed by viewing titles, publication years, and authors' name. Secondly, titles and abstracts of these potentially eligible articles were examined according to predetermined eligibility criteria. Thirdly, full texts were screened and assessed for eligibility. Lastly, the data extraction and quality assessment was conducted. Disagreements and discrepancies were resolved by discussion between two authors (MC, JH). Study selection was conducted and recorded according to the PRISMA protocol (15).

### 2.4. Data extraction and outcomes

Our method for data extraction included recording data related to the first author's name, year of publication, number of studies included (by study design), participants information (such as the age, sex, disease), the intervention (such as dose, follow-up time), the comparison (such as placebo implement), the outcomes or effect sizes (such as mortality, morbidity, sample sizes, variables), and the quality (such as the reliability, bias assessment, heterogeneity).

Detailed information was extracted and presented in the order noted above. The pool of clinical trials was then reviewed by identifying trials contained in the eligible meta-analyses and trials published after which were screened. Finally, we organized the data collected above by removing duplicates and categorized them by different patient populations.

The main outcome of interest was all-cause mortality in different patient specific populations. The secondary outcomes were CVD mortality in patients with CVD, chronic kidney disease (CKD), type 2 diabetes (T2DM) and liver diseases, and cancer mortality in the cancer population.

### 2.5. Quality assessment

The methodological quality of each eligible meta-analysis and systematic review was assessed using A Measurement Tool to Assess Systematic Reviews (AMSTAR), a widely utilized instrument with high reliability, validity and practicality. On the basis of representative assessment tools with reference value, empirical evidence formed in the process of long-term use and expert consensus, AMSTAR has been the first priority of methodological quality assessment for systematic reviews and meta-analyses when conducting umbrella reviews (16). We applied 11 items to identify the methodological quality of each included meta-analysis, with each item ranked with “yes”, “no”, “unclear”, and “partially yes”. Under the assignment of “yes” = 1, “no” = 0, “unclear” = 0, and “partially yes” = 0, the meta-analyses can be divided into “high quality” (9~11), “moderate quality” (5~8), and “low quality” (0~4).

We assessed the evidence certainty of included meta-analyses and systematic reviews with Grading of Recommendations Assessment, Development and Evaluation (GRADE), a tool without the stereotyped restriction of study types, presenting the evidence certainty and assessment considerable objectivity and reliability. The influence factors can be categorized into “study limitation”, “indirectness”, “inconsistency”, “imprecision”, and “publication bias”. Eligible meta-analyses and systematic reviews were classified as “high”, “moderate”, “low”, and “very low” (17).

As for the included updated RCTs not contained within the available meta-analyses and systematic reviews, the Cochrane Risk of Bias (Cochrane ROB) was applied to evaluate evidence reliability. Updated RCTs were categorized as “low risk”, “unclear”, and “high risk” under 7 aspects of bias assessment.

Two authors (MC, JH) independently applied AMSTAR, GARDE, and Cochrane ROB for the quality assessment and made a consensus when studies with discordance were encountered.

### 2.6. Data synthesis and statistical analysis

We created an evidence map presenting the certainty of prior evidence for the effect of vitamin D on all-cause mortality in different diseases via Microsoft Excel 2016. We use the area representing the quality of the included studies in each section, which was evaluated using AMSTAR, GRADE, and The Cochrane ROB.

We implemented a fixed effect model for estimation, and heterogeneity was assessed by I2 statistics. If I2 is >50%, it suggested a high level of heterogeneity, in which we transitioned to implementing a random effect model for a more objective estimate; in order to draw an overall more precise conclusion. We also assessed possible sources of heterogeneity across studies by using subgroup analyses in order to reduce publication bias.

Statistical analyses were conducted using Review Manager (RevMan), version5.4, The Cochrane Collaboration, 2020. The effect measure was risk ratio for the outcome. Significant level was set at 0.05 for all analyses, while which was adjusted to 0.10 when ongoing the Egger regression test for its limited statistical power. Estimates of publication bias were considered using funnel plots if there were more than 10 included studies.

## 3. Results

### 3.1. Search results

**Figure 1:**
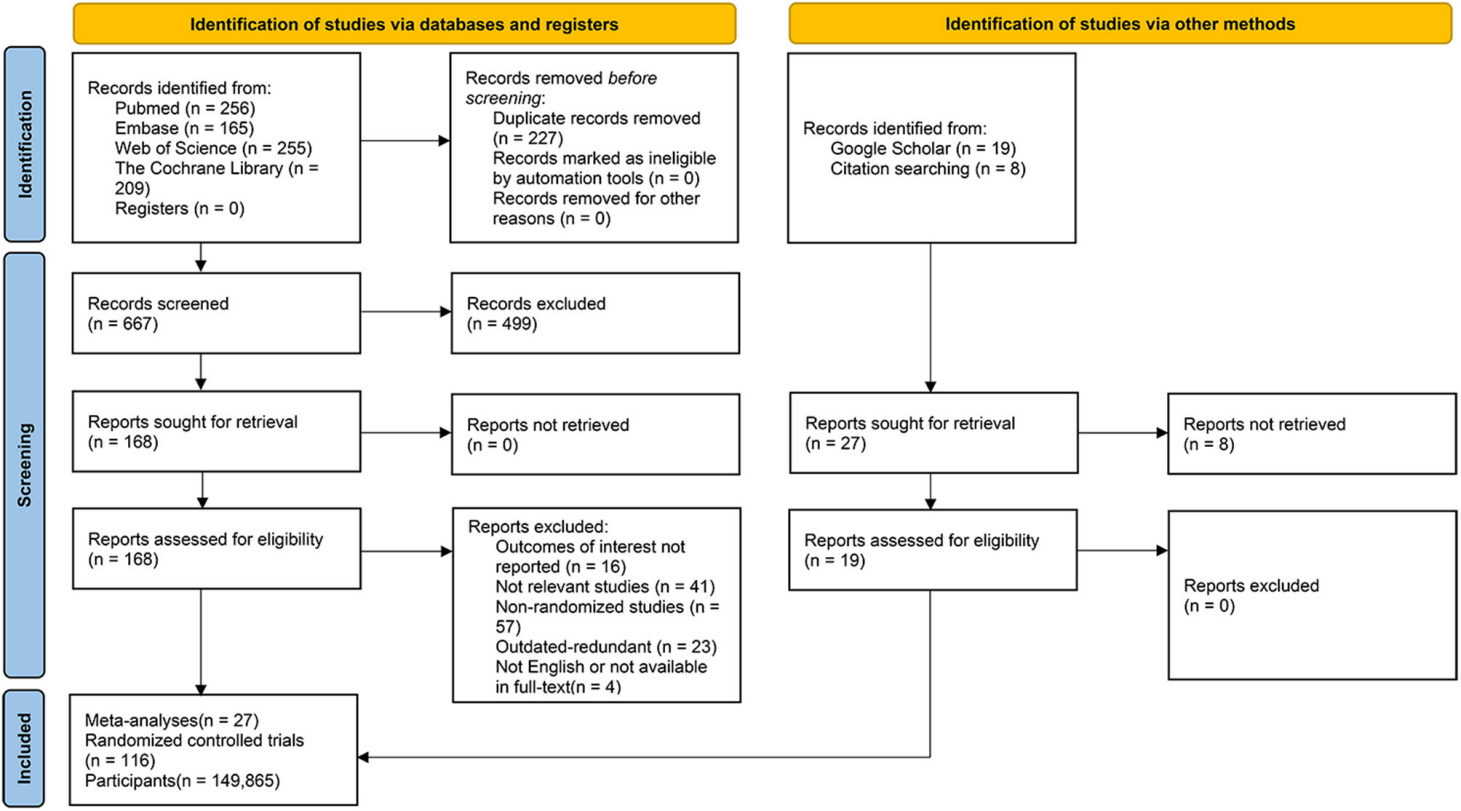
PRISMA flow diagram.

We identified 885 records from database searches. After removing overlapping studies and screening at the title and abstract level, a total of 168 articles were remained for full-text review. Of these 168 studies, 141 articles were excluded, including 16 studies which did not update their outcomes of interest, 41 reviews focused on non-relevant themes, 57 meta-analyses which were composed of non-randomized studies or irrelevant study types (e.g., umbrella review, narrative review, cross-sectional, observational cohort studies et al.), 23 studies which were outdated and redundant (we included the most recent iterations of such studies), as well as 4 articles with language issues in full-text reading or the full-text was not readily accessible. Overall, a total of 27 (7–9, 18–41) meta-analyses were pooled in this umbrella review and 19 updated RCTs newly published were added, with the inclusion of a total of 116 RCTs (10, 42–156) overall comprising 149, 865 participants altogether (Figure 1).

The baseline characteristics of the included RCTs and meta-analyses/systematic reviews in this umbrella review were presented in Supplementary Tables 1, 2.

### 3.2. Quality assessment

Methodological assessment noted that among the 27 eligible meta-analyses and systematic reviews included, 16 ranked “high” level, 9 ranked “moderate” level, 2 evaluated with “low” methodological level (Detailed information of AMSTAR assessment listed in Supplementary Table 3). According to the results of GRADE, the evidence certainty in this umbrella review was classified as 13 “high”, 7 “moderate”, 4 “low” and 3 “very low” (Detailed information of GRADE assessment was listed in Supplementary Table 4). The Cochrane ROB assessment and detailed information of updated RCTs is listed in Supplementary Table 5 and Supplementary Figures 1, 2. Results for the heterogeneity test performed are presented in Supplementary Figures 3–8.

### 3.3. General population

Forty-three citations (10, 42–82) were assessed for the risk of all-cause mortality in general population with vitamin D intervention. Publication dates ranged from 1983 to 2022. The general finding reports that there is no statistically significant difference between the effects of vitamin D and placebo on all-cause mortality in general population (RR, 0.99[95%CI, 0.96 to 1.03]). Subgroup analyses among female, male, and menopausal women populations indicate the same non-significant result. Results were quite consistent among the 43 RCTs, only two reported a harmful effect of vitamin D on all-cause mortality in general population compared with placebo (63, 64) (RR, 3.70[95%CI, 1.06 to 12.92], 1.20[95%CI, 1.04 to 1.37]), while others presented non-significant results (Supplementary Figure 9).

### 3.4. Cancer population

#### 3.4.1. All-cause mortality in cancer population

Nine retrieved RCTs (61, 68, 79, 83–88) were assessed the risk for all-cause mortality in cancer population with vitamin D intervention. Publication dates ranged from 2003 to 2019. Results presents that no significant effects were seen for all-cause mortality in cancer patients treated with vitamin D (RR, 0.93[95%CI, 0.83 to 1.05]). Out of the 9 RCTs, one revealed that vitamin D may increase the all-cause mortality in prostate cancer patients (87) (RR, 1.25[95%CI, 1.04 to 1.50]), while the study lead by Avenell reported an opposite result (84) (RR, 0.78[95%CI, 0.62 to 0.97]) (Supplementary Figure 10).

#### 3.4.2. Cancer mortality in cancer population

**Figure 2:**
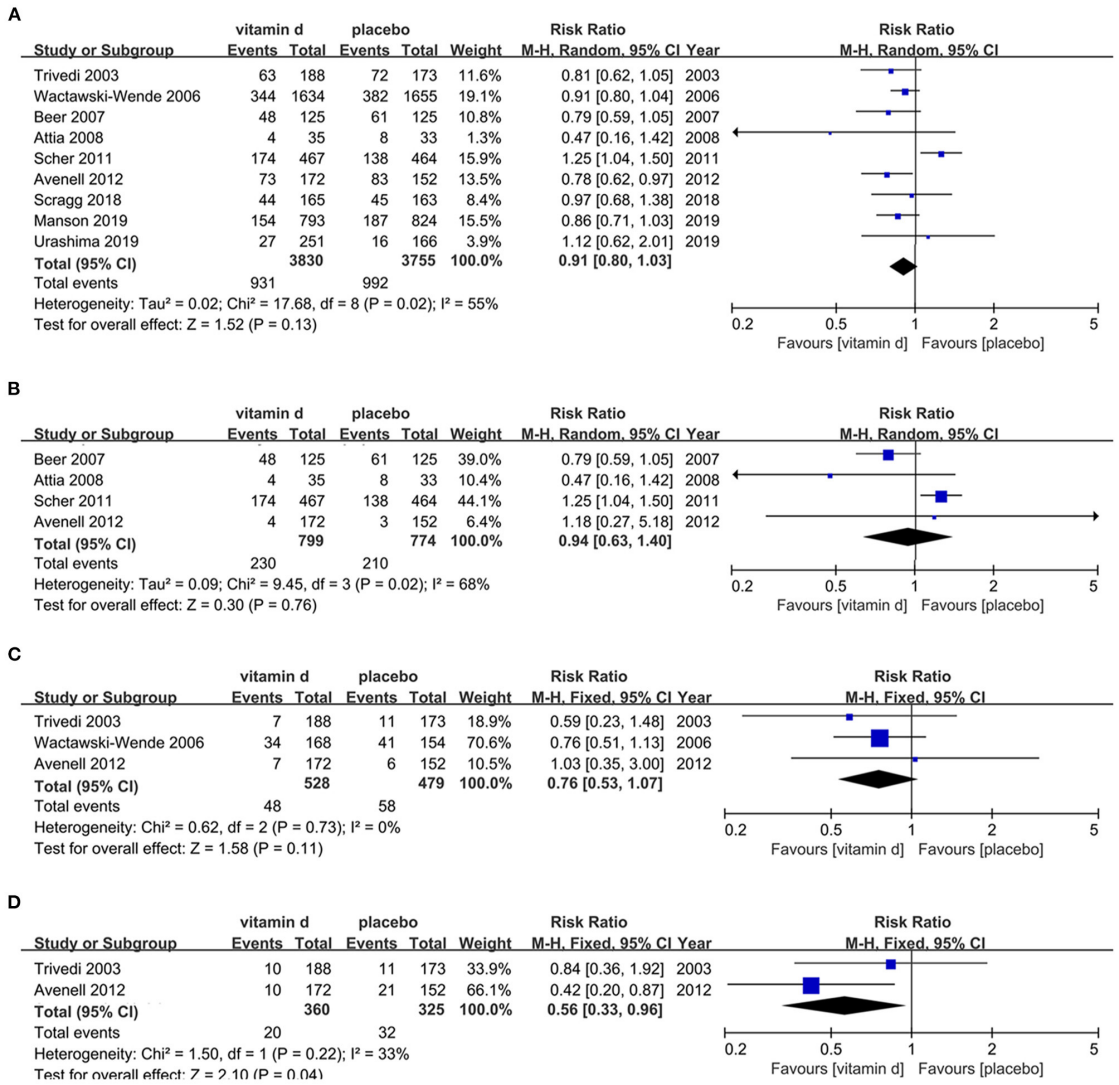
Effects of vitamin D on cancer mortality in cancer population. **(A)** Forest plot showing effects of vitamin D on cancer mortality in all kinds of cancer population. **(B)** Subgroup analysis on prostate cancer mortality in prostate cancer population. **(C)** Subgroup analysis on digestive cancer mortality in digestive cancer population. **(D)** Subgroup analysis on respiratory cancer mortality in respiratory cancer population.

Nine retrieved articles (61, 68, 79, 83–88) were assessed for the risk of cancer mortality in the cancer patient population with vitamin D supplementation. Publication dates ranged from 2003 to 2019. Results shows non-significant difference in cancer mortality between cancer patients treated with vitamin D and placebo (RR, 0.91[95%CI, 0.80 to 1.03]). However, subgroup analyses imply that supplementation of vitamin D was associated with decreased risk of respiratory tract cancer mortality (RR, 0.56[95%CI, 0.33 to 0.96]), while subgroup analyses among prostate cancer and digestive tract cancer patients presented non-significant results (Figure 2).

### 3.5. CKD population

#### 3.5.1. All-cause mortality in CKD population

Twenty-four RCTs (89–112) were cited to estimate the risk for all-cause mortality in CKD population with vitamin D supplement. Publication dates ranged from 1981 to 2021. No statistically significant discrepancy was found in all-cause mortality between vitamin D and placebo supplement, no matter in all stage CKD population (RR, 1.10[95%CI, 0.89 to 1.34]) nor late-stage patients (dialysis-dependent patients) (RR, 1.09[95%CI, 0.88 to 1.34]) (Supplementary Figures 11A, B).

#### 3.5.2. CVD mortality in CKD population

Nine retrieved (92, 95–98, 101, 103, 107, 109) studies were evaluated for risk of CVD mortality in CKD population treated with vitamin D. Publication dates ranged from 1995 to 2015. All included citations noted common conclusions, that no differences were shown in this outcome regardless of the stage of CKD (all stage CKD population: RR, 1.20[95%CI, 0.52 to 2.75]; dialysis population: RR, 0.80[95%CI, 0.39 to 1.65]) (Supplementary Figures 11C, D).

### 3.6. Respiratory disease population

**Figure 3:**
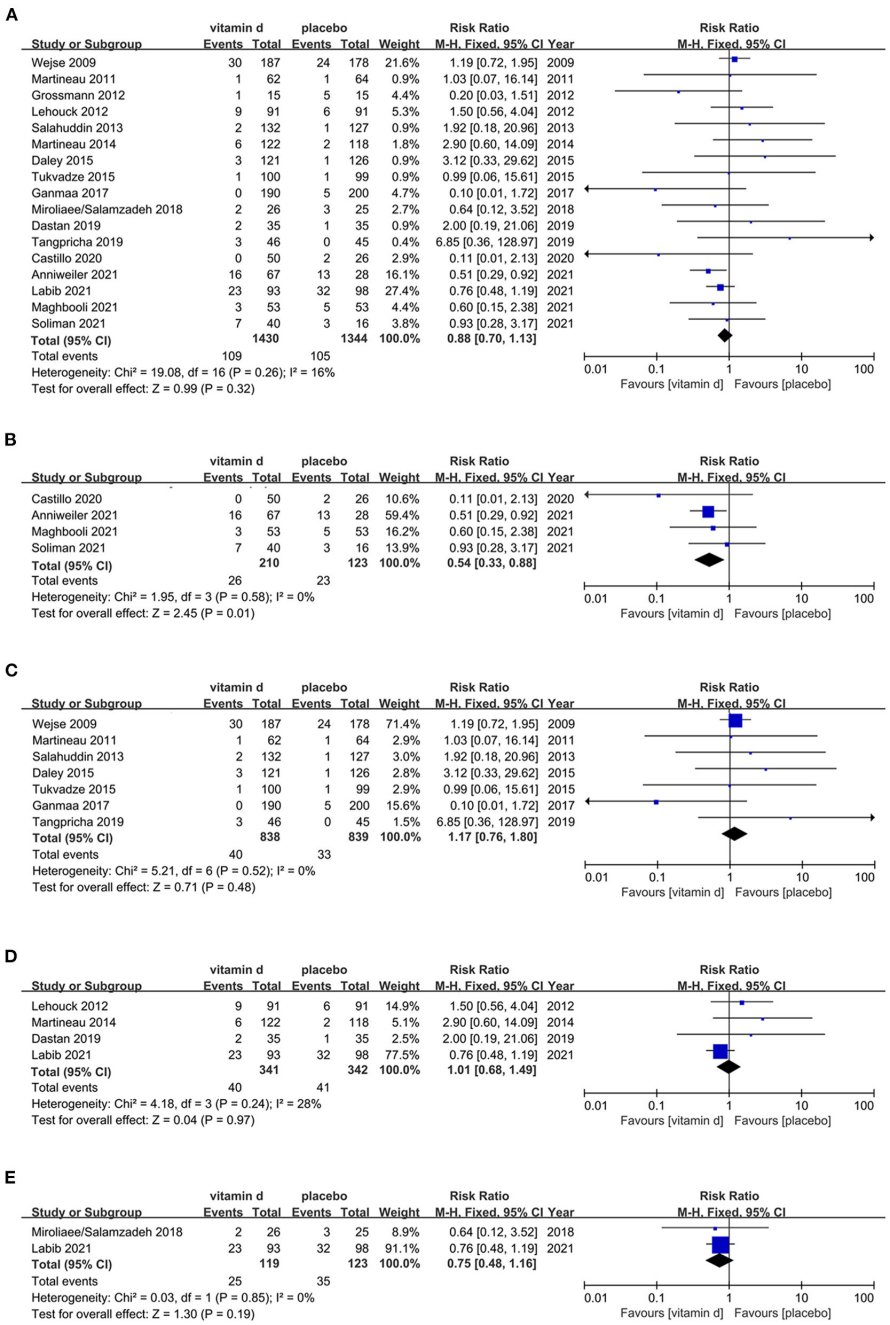
Effects of vitamin D on all-cause mortality in respiratory disease population. **(A)** Forest plot showing effects of vitamin D on all-cause mortality in all kinds of respiratory diseases population. **(B)** Subgroup analysis on all-cause mortality in COVID-19 population. **(C)** Subgroup analysis on all-cause mortality in PTB population. **(D)** Subgroup analysis on all-cause mortality in COPD population. **(E)** Subgroup analysis on all-cause mortality in pneumonia population.

A total of 17 RCTs (113–129) were estimated for the risk of all-cause mortality in respiratory diseases patients with vitamin D supplementation. Publication dates ranged from 2009 to 2021. The analyze results shows that there's no statistically significant difference between vitamin D and placebo among respiratory diseases population (RR, 0.88[95%CI, 0.70 to 1.13]). However, subgroup analysis indicates that vitamin D may reduce the all-cause mortality among COVID-19 patients (RR, 0.54[95%CI, 0.33 to 0.88]). Other respiratory diseases including pulmonary tuberculosis (PTB), chronic obstructive pulmonary diseases (COPD) and pneumonia, subgroups analyses all present irrelevant results (Figure 3).

### 3.7. Critically ill population

A total of 13 studies (114, 124, 130–140) were retrieved. Publication dates ranged from 2014 to 2020. No significant associations was observed about the risk of all-cause mortality in critically ill patients with vitamin D intervention (RR, 0.93[95%CI, 0.81 to 1.07]). Furthermore, according to the subgroup analyses, regardless of the follow-up time (7d, 30d, 90d, 180d) nor the severity of the illness (ICU stay, hospitalization), the supplement of vitamin D has nothing to do with affecting all-cause mortality in critically ill patients (Supplementary Figure 12).

### 3.8. CVD population

#### 3.8.1. All-cause mortality in CVD population

In this part of the review, which was based on 8 RCTs (68, 78, 79, 108, 141–144), vitamin D did not demonstrate any clinically significant differences in all-cause mortality in CVD patients compared with placebo (RR, 1.03[95%CI, 0.91 to 1.18]). Publication dates ranged from 2003 to 2019 (Supplementary Figure 13A).

#### 3.8.2. CVD mortality in CVD population

Five studies (68, 78, 79, 108, 144) were assessed in this section. Publication dates ranged from 2003 to 2019. No significant association between vitamin D supplementation and CVD mortality in CVD population was reported among the 5 included RCTs (RR, 1.03[95%CI, 0.90 to 1.18]) (Supplementary Figure 13B).

### 3.9. Musculoskeletal disease population

Six citations (84, 145–149) were evaluated the risk for all-cause mortality in musculoskeletal disease patient population with vitamin D supplementation. Publication dates ranged from 1973 to 2016. No clinically significant relevance was demonstrated in this outcome (RR, 0.97[95%CI, 0.87 to 1.08]). The subgroups of osteoarthritis (OA), rheumatoid arthritis (RA) and post-fracture operation also reported non-significant results (Supplementary Figure 14).

### 3.10. Sepsis population

Four identified RCTs (136, 139, 150, 151) has been tested for the effect of vitamin D on all-cause mortality in the sepsis patient population. Publication dates ranged from 2014 to 2021. On the basis of this analysis, there's no therapeutic nor harmful effect of vitamin D treatment on all-cause mortality in sepsis population (RR, 0.82[95%CI, 0.58 to 1.15]) (Supplementary Figure 15).

### 3.11. T2DM population

#### 3.11.1. All-cause mortality in T2DM population

Three interventions (96, 152, 153) were evaluated the risk for all-cause mortality in T2DM population with vitamin D intervention. Publication dates ranged from 2010 to 2016. Result demonstrated that vitamin D intervention has no association with all-cause mortality in T2DM patients (RR, 0.80[95%CI, 0.21 to 2.97]) (Supplementary Figure 16A).

#### 3.11.2. CVD mortality in T2DM population

Two studies (96, 153) evaluated the risk for CVD mortality in the T2DM population with vitamin D intervention. Publication dates ranged from 2010 to 2012. Both studies did not demonstrate any statistically significant findings for this outcome (RR, 1.00[95%CI, 0.14 to 7.09]) (Supplementary Figure 16B).

### 3.12. Liver disease population

#### 3.12.1. All-cause mortality in liver disease population

**Figure 4:**
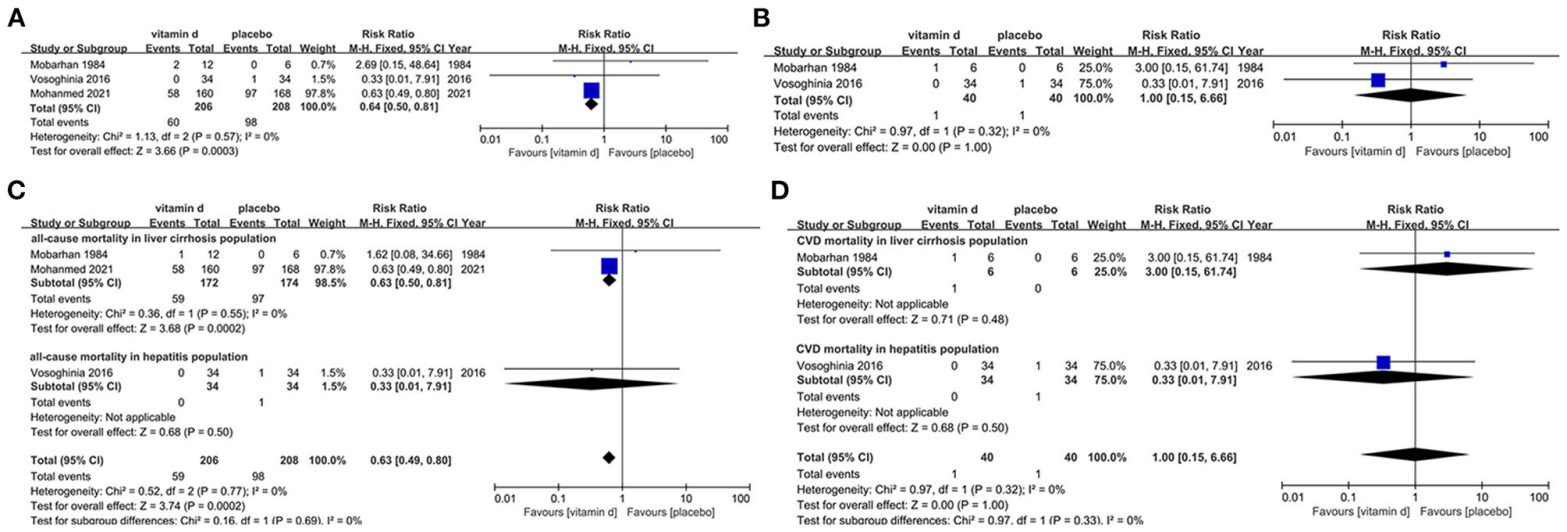
Effects of vitamin D on mortality in liver disease population. **(A)** Forest plot showing effects of vitamin D on all-cause mortality in all kinds of liver diseases population. **(B)** Forest plot showing effects of vitamin D on CVD mortality in all kinds of liver diseases population. **(C)** Subgroup analysis on all-cause mortality in liver cirrhosis and hepatitis population. **(D)** Subgroup analysis on CVD mortality in liver cirrhosis and hepatitis population.

Only 3 studies (154–156) matched our inclusion criteria and were assessed for the risk of all-cause mortality in liver diseases population with vitamin D supplementation. Publication dates ranged from 1984 to 2021. In this section, it implies that vitamin D supplement shows statistically significant reduction of all-cause mortality in liver disease patients (RR, 0.64[95%CI, 0.50 to 0.81]), which suggests that vitamin D might be beneficial in the liver disease patient population. Especially for cirrhotic patients, vitamin D may be therapeutic (RR, 0.63[95%CI, 0.50 to 0.81]), however this benefit may not be as applicable in hepatitis patients (Figures 4A, B).

#### 3.12.2. CVD mortality in liver disease population

Two studies (154, 156) were evaluated for the risk of CVD mortality in the liver disease population with vitamin D intervention. According to the analysis result, vitamin D supplementation is not significantly associated with risk for this outcome regardless of the severity of the exacerbation of liver disease (RR, 1.00[95%CI, 0.15 to 6.66]) (Figures 4C, D).

### 3.13. Evidence map

**Figure 5:**
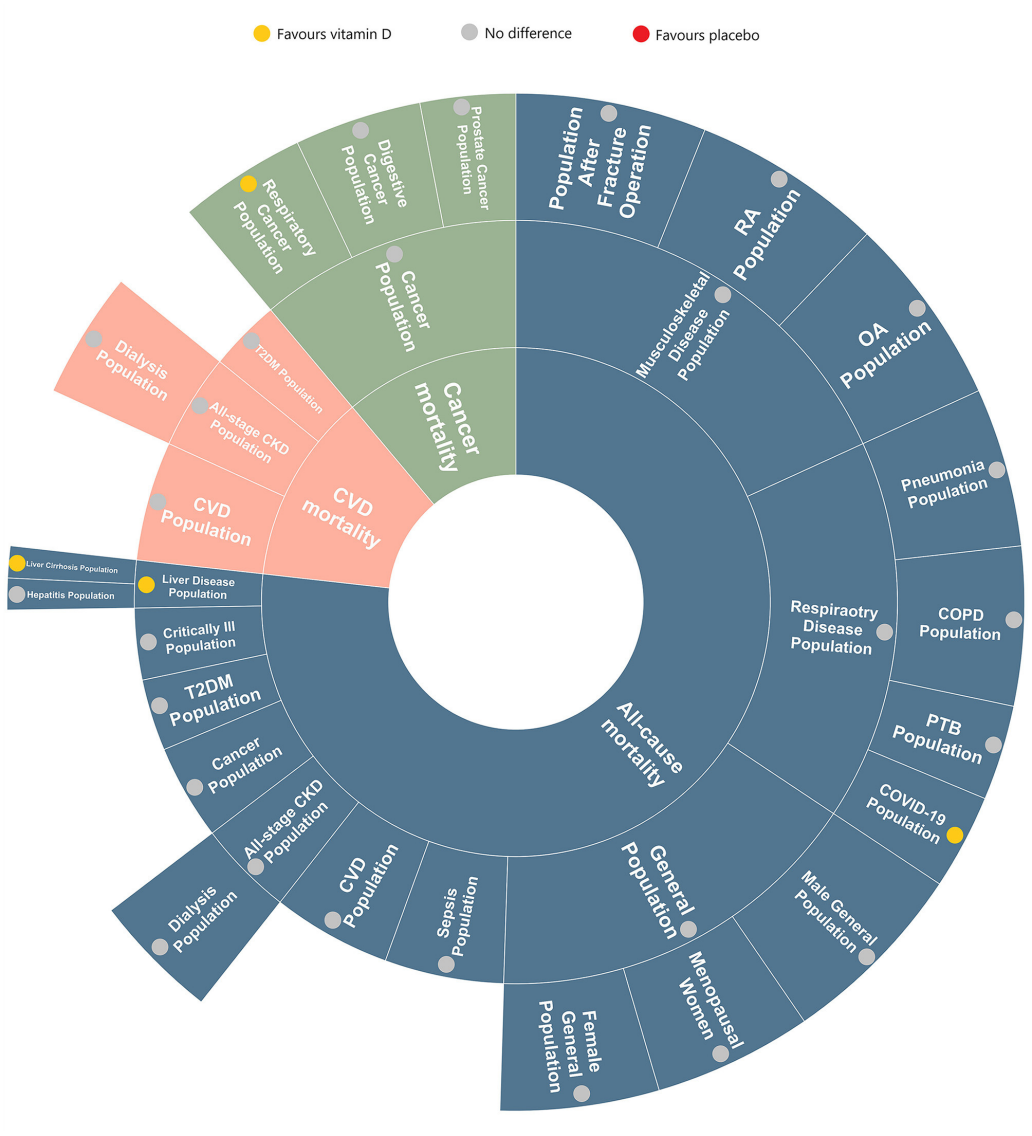
Evidence map of umbrella review by participants and mortality.

Figure 5 is an evidence map summarizing the effects of vitamin D on all-cause mortality in different diseases amongst the included RCTs in our review. The area of different section in this map represents the quality of the articles (based on the standardized mean scores of AMSTAR, GRADE, and The Cochrane ROB) related to the disease. For example, in the quality assessment, the standardized mean score of critically ill population is 3, while which in general population is 5, so the latter has a larger area than the former.

Most of the analyzed results implies that vitamin D has no beneficial nor harmful effect on all-cause mortality in certain populations. However, a portion of data shows that vitamin D plays a therapeutic role in reducing respiratory cancer mortality in respiratory cancer patients and all-cause mortality in COVID-19 and liver diseases population (especially for cirrhotic patients). Due to the limitation of available RCTs, there was a paucity of data evaluating the impact of vitamin D on all-cause mortality in the T2DM population, sepsis population and liver disease population, which remains to be discovered and corrected.

## 4. Discussion

Based on this evidence map and umbrella review of 27 meta-analyses and 116 RCTs with 149, 865 participants, we found that the effect of vitamin D on mortality varies depending on different health condition. Our analysis suggests that vitamin D may reduce respiratory cancer mortality in patients with respiratory cancer, as well as all-cause mortality in patients with COVID-19 or liver diseases (especially cirrhosis). However, vitamin D can't reduce cancer mortality in cancer patients or all-cause mortality in the populations with health conditions such as cancer, CKD, respiratory diseases (COVID-19, PTB, COPD, and pneumonia), critical illness, CVD, musculoskeletal diseases (OA, RA, and post-fracture surgery), sepsis, T2DM, and hepatitis.

Comparing our evidence with previous studies, some inconsistency did exist. On the one hand, our results shows that vitamin D has a therapeutic effect on mortality in cirrhotic, respiratory cancer and COVID-19 patients. However, there is still disagreement in the literature. In hepatic patients, Bjelakovic et al. (41) reported that vitamin D has non-significant effects on mortality in cirrhosis, which was questioned by Mohamed (155) with a therapeutic result, consistent with our finding. In cancer patients, divergent opinions are even greater. A reduced cancer mortality after vitamin D supplementation was reported by a meta-analysis basing on 74,655 participants in 2019 (9). However, a subsequent large-scale RCT study pointed out that higher cancer mortality may present after excluding the first 2 years of follow-up (10). What's more, since COVID-19 outbreak, the effectiveness of vitamin D as a treatment has been widely discussed. Four RCTs evaluated mortality with vitamin D supplementation, and only one flaw-designed RCT showed a weak benefit of vitamin D treatment on all-cause mortality in COVID-19 patients (114), which is consistent with our findings. The other RCTs reported similar results between vitamin D and placebo groups (114, 121, 126). On the other hand, we found no significant differences between vitamin D and placebo in the other population groups we studied. Most available studies support our perspective, but a 2014 meta-analysis on vitamin D supplementation's effect on all-cause mortality in the general population showed inconsistencies upon further review (7).

Reasons behind these discrepancies in vitamin D supplementation efficacy require further consideration. The top of which regards to follow-up time, which indicates the onset and duration of drug effectiveness. Some studies excluded the first 2 years of follow-up analysis, assuming that vitamin D might not have reached an adequate onset time in first 2 years (10, 68). On the contrary, other studies suggested that the effects of vitamin D may disappear within one or two years after the cessation of supplementation (84). These findings raise concerns about the accuracy and reliability of included trials in our review. Due to the chronic nature of many diseases in this review, a significant induction period may be required to capture outcomes (1). Insufficient follow-up time may only reveal changes in progression rather than final outcomes, leading to discrepancies in mortality that are difficult to analyze. Based on this situation, the null hypothesis is usually not rejected due to the high possibility of type 2 error Thus, different follow-up times may be one of the reasons of the inconsistency.

Moreover, the variation in baseline serum vitamin D level may be another reason of the inconsistency, which could affect appropriate supplementation doses and any potential impact on mortality. The baseline vitamin D level in the RCTs included in our review range from 128.7 ± 47.3 nmol/L (76) to 16.4 ± 5.2 nmol/L (140), with daily supplement doses varying from 600,000 IU (60) to 800IU (65). It's acknowledged that sun exposure promotes the cutaneous vitamin D3 formation (157), therefore different levels of sun exposure and different health conditions lead to varying vitamin D metabolic status, resulting in different serum 25(OH)D levels. Thus, different doses of vitamin D supplementation are required to achieve protective effects (158). Some investigators suggested that the protective vitamin D concentration is 75–110 nmol/L, to achieve such a level, about 1,800–4,000 IU per day is needed orally. That is to say, a low dose (< 800 IU/d) may not be sufficient for protection (77, 159). Nevertheless, high-dose vitamin D supplementation also requires prudent consideration. Some argued that high vitamin D concentrations may be harmful (160), while others suggested that only high doses have positive effects. Based on this, there is an urgent need to find the best dosage for optimal treatment outcomes. However, no study has identified the ideal vitamin D supplementation dose that caters to all health conditions. The pathological heterogeneity results in different recommended doses for different diseases. For respiratory infections, a daily supplementation of 400–1,000 IU is most effective (161), and osteoporosis patients are advised to take 800 IU/day (162), while primary hyperparathyroidism requires a higher dose of 2,800 IU/day due to the low serum calcium trait of PTH (163). Therefore, a universal agreement has been reached that the key to supplementing vitamin D is achieving optimal serum 25(OH)D levels, rather than chasing the best supplementary dose. The goal of vitamin D supplementation should be to raise abnormal serum levels back to normal, instead of simply providing a fixed dose. A serum vitamin D level of at least 20 ng/ml is generally considered ideal for most health conditions as recommended (164). However, the common intervention of RCTs is giving a fixed dose, instead of achieving a fixed level, which likely to result in a situation of sufficient vitamin D baseline supplemented with low dose of vitamin D (165), leading to inconsistent results. Additionally, the administration route of vitamin D is also a concern for its effects. The common ways of supplementing vitamin D are oral intake and intramuscular injection. Studies suggest that for general population, oral supplementation is better for loading, while the two routes seem equally effective for maintenance therapy (166). Thus oral administration may be a safer way to take supplementation. What's more, the relationship between serum vitamin D level and disease occurrence is complex. Some suggest that low levels of serum vitamin D may be the cause of various diseases, while others argue it as the consequence of disease. Amiel et al. (167) pointed out that vitamin D deficiency is tightly linked with the susceptibility of SARS-CoV-2, while Smolders et al. (168) alludes that decreased vitamin D status is just the consequence of systematic inflammation in COVID-19. Besides, Bolland et al. (8) presented the idea of “reverse causality”, that low serum vitamin D level is not a consequence of health problems, but rather a cause of various diseases occurring due to reduced sun exposure. However, this viewpoint has been opposed by others based on the theory of statistical type 2 error (169). In this case, vitamin D supplementation might be futile if the vitamin D deficiency is just a consequence of diseases instead of the cause. Based on our umbrella review and evidence map, it's clear that vitamin D supplementation remarkably reduces respiratory cancer mortality. Numerous large-scale observational studies also support the therapeutic superiority of vitamin D for cancer (170, 171). The mechanisms behind that require deep consideration. Many studies have shown that vitamin D has immune-enhancing effects, boosting the immune system by activating specific immune cells for resisting viruses, bacterium, and tumor cells. This theory supports our findings in cancer mortality. Current explanation mostly supports that vitamin D affects gene expression in two ways: long-term by binding to specific gene promoters and modulating protein expression related to cell differentiation and growth, such as CYP27B1 (172, 173), and short-term by directly affecting VDR without involving protein production through The Central Law (174, 175). Both mechanisms can modify the inflammatory response and alter the tumor microenvironment, resulting in an immunological effect (176). Research published in 2020 suggested that the gene regulation modulated by vitamin D is not only evident in patients, but also in tumor cells. Vitamin D may be involved in the re-programming and adhesion-modifying of tumor cells, and result in better evasion of immune surveillance (177, 178). This paradox needs more comprehensive studies to explain.

These mechanisms were also explored of the reduced all-cause mortality in liver diseases. Scientific f research has revealed a high prevalence of vitamin D deficiency in liver diseases, particularly cirrhosis, which significantly impacts the mortality of patients with hepatic disorders (179–181). The first hydroxylation of vitamin D occurs in the liver, which is crucial for its absorption and activation in the body. Therefore, cirrhosis-related complications like portal hypertension can hinder the conversion of vitamin D to its active form (179). What's more, patients with hepatic disorders often lack bile salts, which are necessary for absorbing fat-soluble vitamins in the gastrointestinal tract (181, 182). With the function of infection preventing, angiogenesis influencing, apoptosis modulating, and differentiation and proliferation affecting (179), vitamin D supplementation has therefore become a therapy to improve liver cirrhosis. It was widely acknowledged that β-catenin plays a crucial role in fibrogenesis. In 2018, it was confirmed that vitamin D can silence the Wnt1/β-catenin pathway, which suppresses the activation of hepatic stellate cells and reduces collagen fiber secretion, leading to the inhibition of type I/III collagen formation, thereby ameliorating the deterioration of liver cirrhosis (183). In addition, the polymorphism of VDR in immune cells and hepatocytes also presents a high correlation with liver cirrhosis, owing to the co-mediation of VDR and bile salts for vitamin D uptake (181, 184, 185). More importantly, supraphysiological concentrations of 25(OH)D3 resulting from artificial vitamin D supplementation can act as a VDR agonist (186), inhibiting Hedgehog (Hh) pathway, reducing the production of hepatitis C virus (HCV) largely, blocking an important predisposing factor for liver cirrhosis (187). Vitamin D is able to directly combine with smoothened released by the combination of Hh and patched, thus suppressing the transcription factor glioma-associated, impeding the replication of HCV effectively and improving sustained virologic responses in patients (188). These findings corroborate and cater to the result of our subgroup analysis on liver cirrhosis.

Based on our results, vitamin D has been shown to reduce all-cause mortality in SARS-CoV-2 infection. The available beneficial mechanisms of vitamin D supplementation can be broadly divided into the effects on hosts and effects on viruses. On the one hand, vitamin D coordinates hosts' immune system, the angiotensin converting enzyme 2 (ACE2) expression and the renin-angiotensin (RAS) system to resist SARS-CoV-2 (189, 190). Vitamin D can activate VDR on cell membranes to awake immune response (189). High levels of 25(OH)D3 combine with vitamin D-binding proteins in monocytes, facilitating its spread via the bloodstream and increasing the likelihood of binding to VDR. This induces autophagy, where autophagosomes act as antigen presenting stimulants and induce the adaptive immunity (191, 192). What's more, vitamin D also decreases ACE2 expression, effectively blocking COVID-19 entry (189). Moreover, vitamin D supplementation reduces damage to the host by decreasing RAS activity through inducing ACE2/Ang1-7 pathway (189, 193, 194). On the other hand, vitamin D can also hinder the adsorption, penetration and replication of SARS-CoV-2 by inducing the release of cathelicidin, defensins, and soluble ACE2 (195–199). Researchers also noted that the timing of the first vitamin D supplement is crucial for its effectiveness against COVID-19. If taken too late after symptoms appear, it may be ineffective in reducing virus viability and preventing organ damage caused by cytokine storms (200). Generally, observational studies and meta-analyses suggest that vitamin D may be beneficial for COVID-19 (201–203) and deficiency is commonly observed. However, with only a few trials conducted, our knowledge of vitamin D therapy for SARS-CoV-2 may just be the tip of the iceberg. More and better experimental investigations are urgently needed to guide the clinical treatment in the future.

Although our umbrella review found no negative effects of vitamin D on mortality, we did observe some adverse events during the intervention. One common adverse event was kidney stone occurrence, especially when vitamin D treatment was combined with calcium, which was not only occurred in CKD patients, but also reported in patients with cancer (28, 53, 61, 62, 152, 204–207), CVD (20, 208–210), and even respiratory diseases (211). However, some studies suggested that combining vitamin D and Ca2+ may offer greater protection against cancer than using vitamin D alone (212), but the potential benefits of this combination for preventing cancer and the risks of kidney stones are still uncertain. Rigorous trials are needed to provide solid evidence of its effectiveness in future clinical treatments.

**Figure 6:**
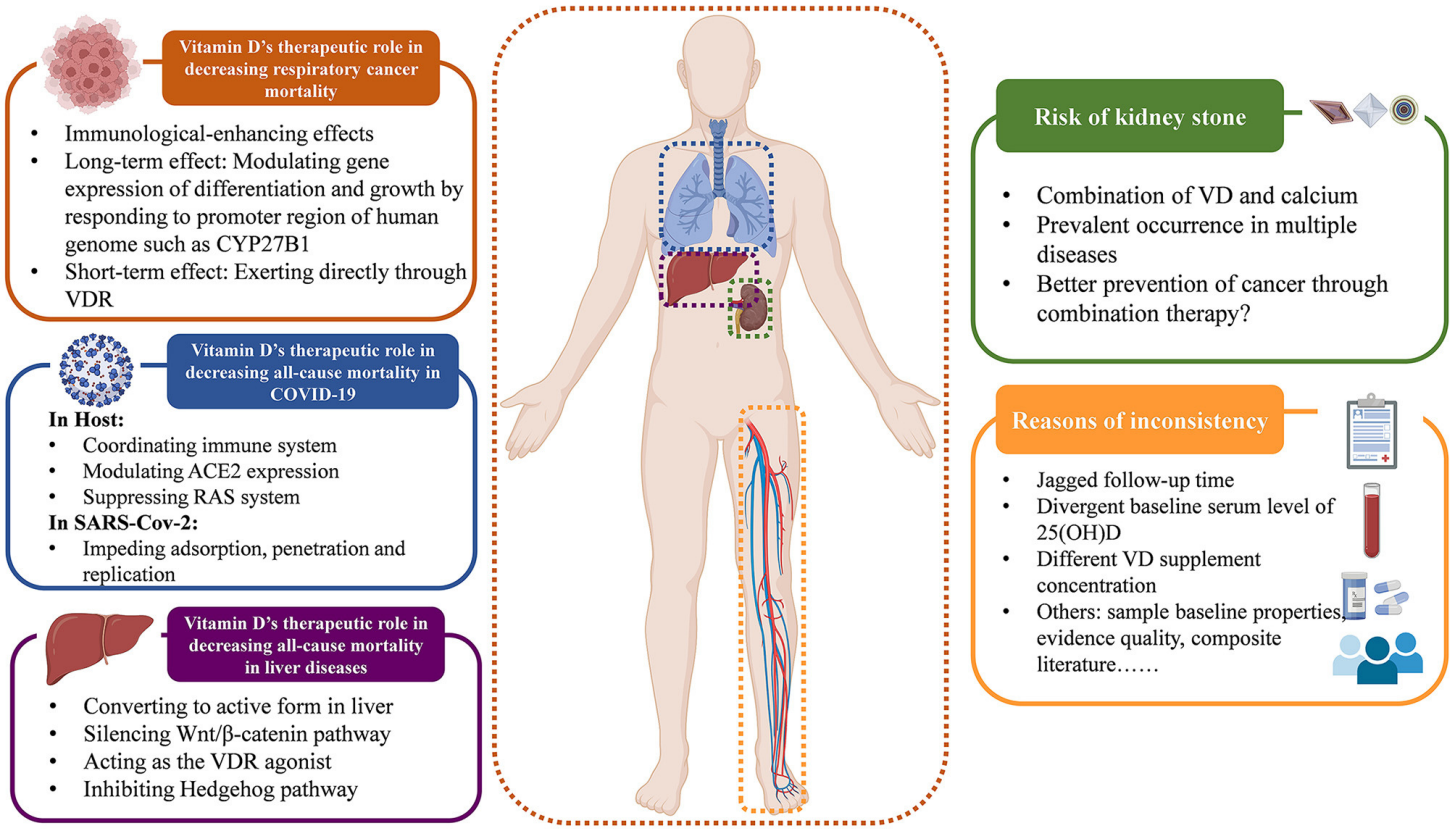
Overall discussion.

We summarize the relevant opinions discussed above in a figure for easy reading in Figure 6.

Nevertheless, our review also has a certain number of limitations. The inherent secondary limitations of the included articles is one of the shortcomings that cannot be avoided. Some meta-analyses with a small range of trials leave the quality uncertain and the result skeptical. Although umbrella review is the top evidence to draw integrated conclusions nowadays, we still need to consider the inherent disadvantages of baseline studies. Secondly, the available RCTs of some focus in this review are still lacking (e.g., only three RCTs were retrieved for liver disease), the insufficiency of baseline studies is bound to impede the analysis and exploration in some populations, resulting in poor data capacity and overall results quality. Moreover, the wide range of vitamin D dosing amount is also one of the limitations that cannot be neglected. We aim to find out the effect of vitamin D on all-cause mortality, thus we prefer not to set boundaries on vitamin D type (vitamin D2, vitamin D3 et al.), intake method (oral intake, muscle injection et al.), combination use (calcium et al.), supplementation concentration, and dosing frequency, which inevitably introduce bias into our research. Besides we didn't focus on the variations in serum vitamin D level (213–215) or the differences in VDR expression among different races, which may affect the effects of vitamin D supplements and introduce bias into our results (216). Last, the comprehensiveness of included diseases' types is faulty. We manually excluded the data of Parkinson (217) and AIDS (218), because these data have not meet the statistical criteria, leaving the results a tiny flaw.

## 5. Conclusions

In summary, this evidence map and umbrella review suggests that vitamin D may reduce respiratory cancer-related mortality in respiratory tract cancer patients, and decrease all-cause mortality in COVID-19 or liver disease population (particularly in liver cirrhosis patients). However, there is no evidence to support the beneficial or harmful effects of vitamin D on all-cause mortality and other specific-cancer mortality in other health conditions. Simultaneously, this study may provide clinicians a statistical foundation to adjust their vitamin D supplementation regimen for different health conditions. However, due to the discrepancy in follow-up time and inadequate RCTs, there is a clear need for better designed trials and further studies to draw a more convincing conclusion on the role of vitamin D in the future.

## Data availability statement

The original contributions presented in the study are included in the article/Supplementary material, further inquiries can be directed to the corresponding author.

## Author contributions

MC and CH performed the data collection. MC conducted the statistical analysis of all data. MC and JH drafted and edited the original manuscript and participated in the conception and design of the original study. MG and SW revised the manuscript and participated the original study design. All authors read and approved the final manuscript.
