# Supplementary material for: The effects of vitamin D on all-cause mortality in different diseases: an evidence-map and umbrella review of 116 randomized controlled trials
Source: Front Nutr. 2023 Jun 22;10:1132528. doi: 10.3389/fnut.2023.1132528 (PMC10325578; doi:10.3389/fnut.2023.1132528)
Supplement: Supplementary file 1 [file Data_Sheet_1.docx]

Supplementary Material

The effects of vitamin D on all-cause mortality in different diseases: an evidence-map and umbrella review of 116 randomized controlled trials

Mingyu Cao, MD, Chunrong He, Matthew Gong, Song Wu, Jinshen He*

*** Correspondence:** Jinshen He: Jinshen.he@hotmail.com

# Supplementary Tables and Figures

## Supplementary Tables

**Supplementary Table 1.** Baseline characteristics of the included RCTs

**Supplementary Table 2.** Overview of the included meta-analyses/systematic reviews

**Supplementary Table 3.** AMSTAR assessment

**Supplementary Table 4.** GRADE assessment

**Supplementary Table 5.** The Cochrane ROB assessment

## Supplementary Figures


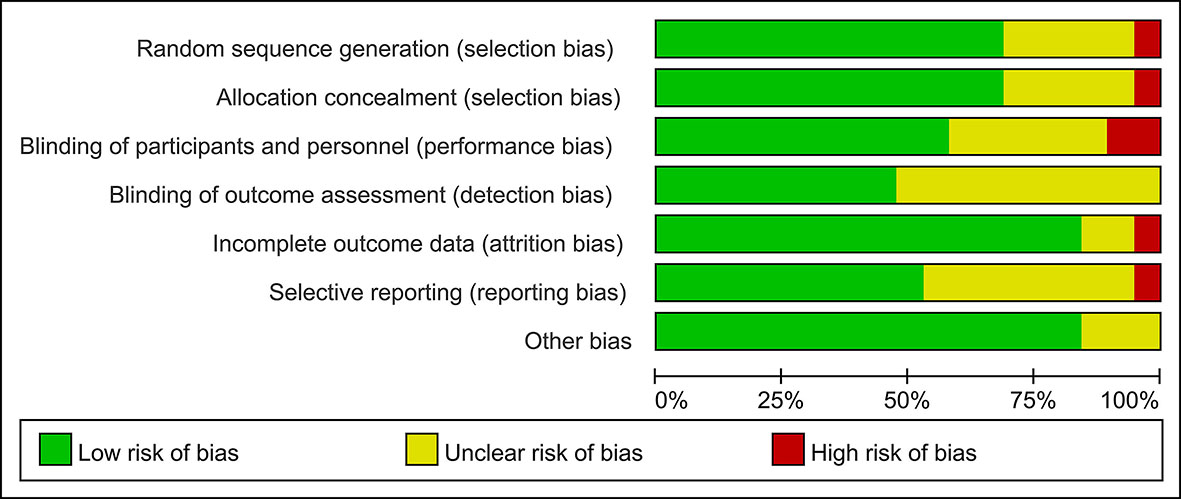


**Supplementary Figure 1.** Risk of bias summary


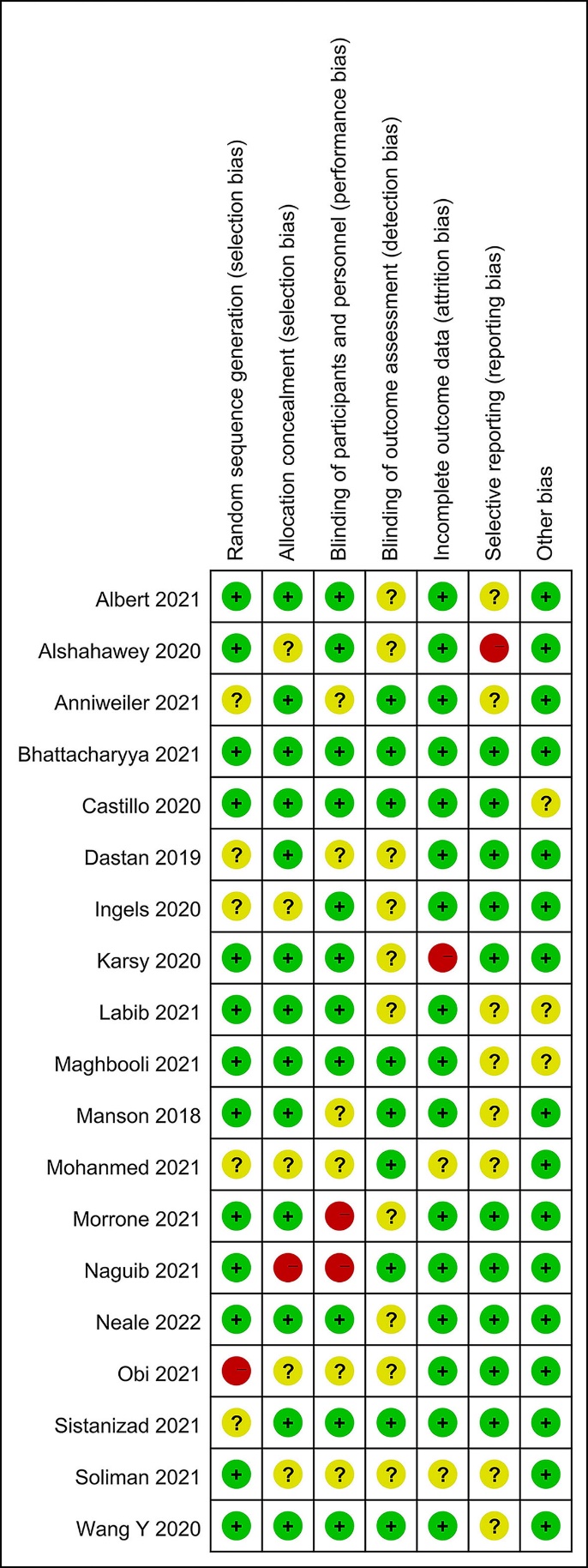


**Supplementary Figure 2.** Risk of bias graph

**
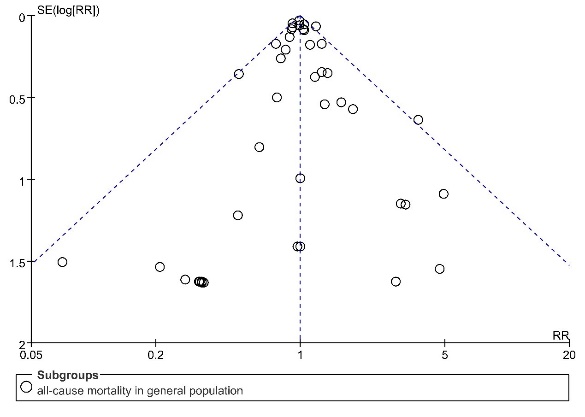
**

**Supplementary Figure 3.** Funnel plot showing heterogeneity of all-cause mortality in general population

**
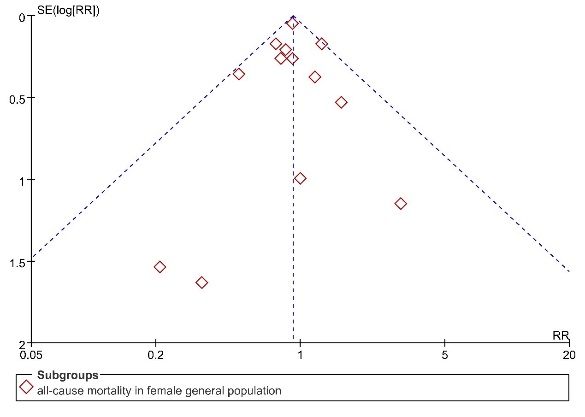
**

**Supplementary Figure 4.** Funnel plot showing heterogeneity of all-cause mortality in female general population

**
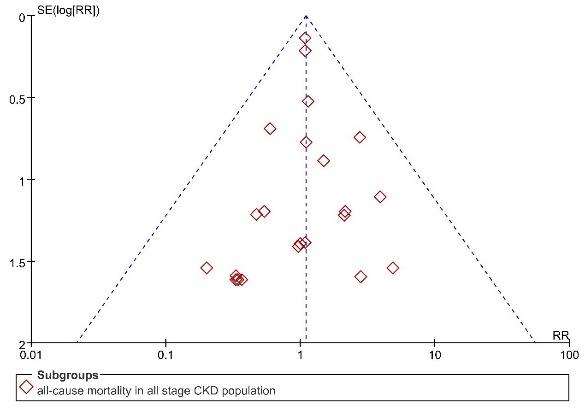
**

**Supplementary Figure 5.** Funnel plot showing heterogeneity of all-cause mortality in all-stage CKD population

**
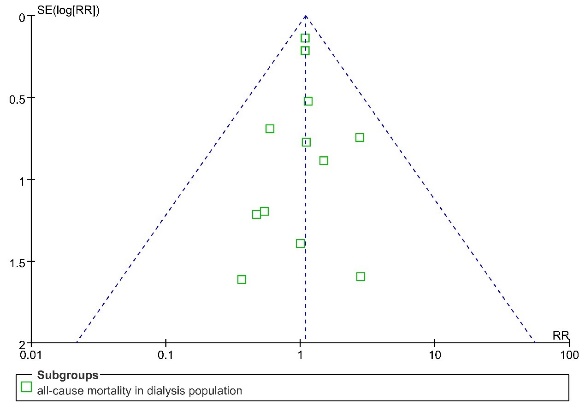
**

**Supplementary Figure 6.** Funnel plot showing heterogeneity of all-cause mortality in dialysis population

**
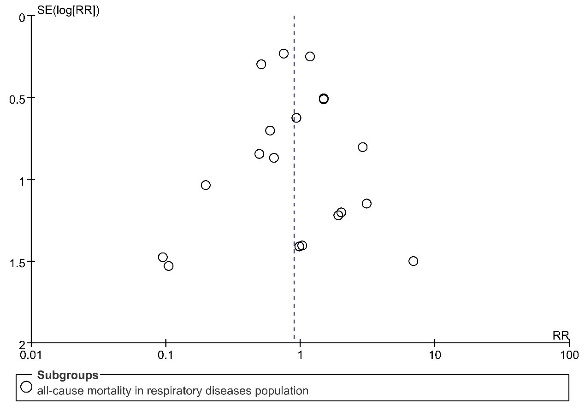
**

**Supplementary Figure 7.** Funnel plot showing heterogeneity of all-cause mortality in respiratory disease population

**
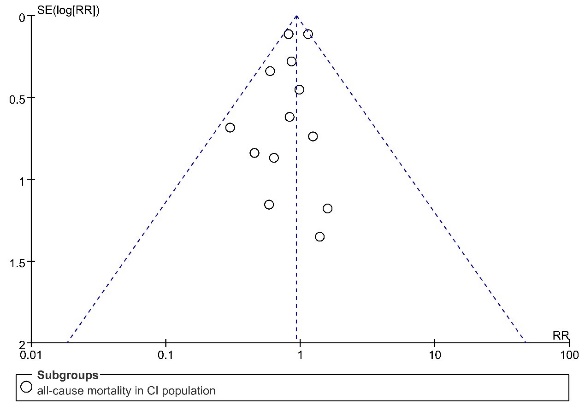
**

**Supplementary Figure 8.** Funnel plot showing heterogeneity of all-cause mortality in critically ill population (Abbreviation: CI-critically ill)
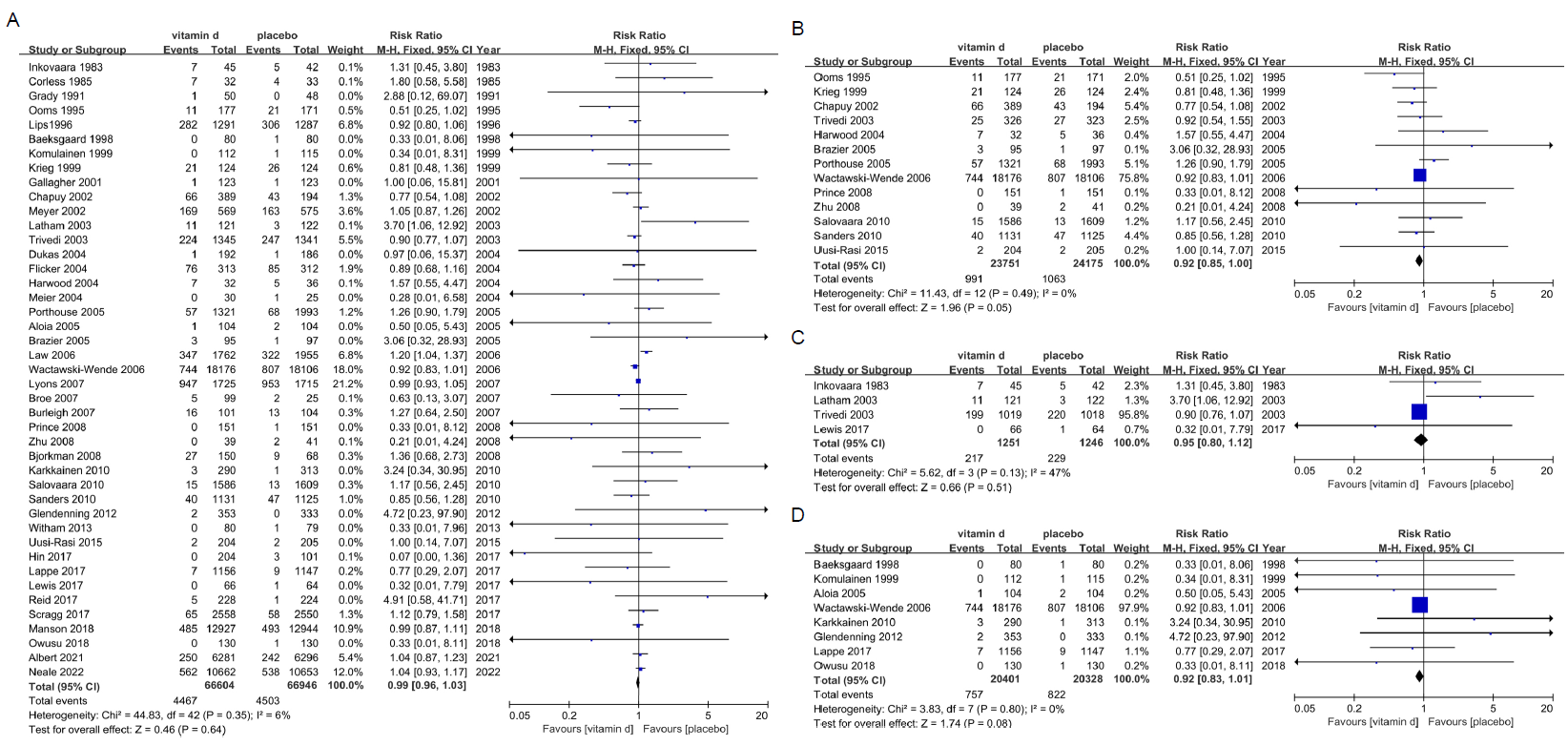
**Supplementary Figure 9.** Effects of vitamin D on all-cause mortality in general population. (A): Forest plot of effects of vitamin D on all-cause mortality in general population. (B): Subgroup analysis of all-cause mortality in female population. (C): Subgroup analysis of all-cause mortality in male population. (D): Subgroup analysis of all-cause mortality in menopausal women.


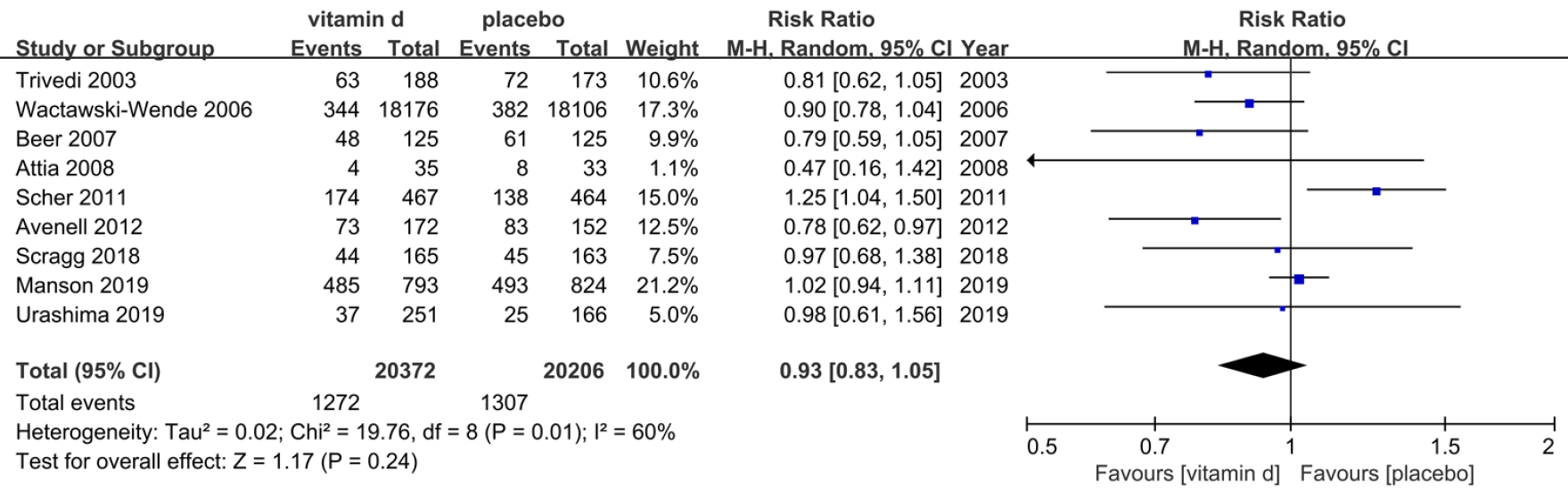


**Supplementary Figure 10.** Forest plot showing effects of vitamin D on all-cause mortality in cancer population


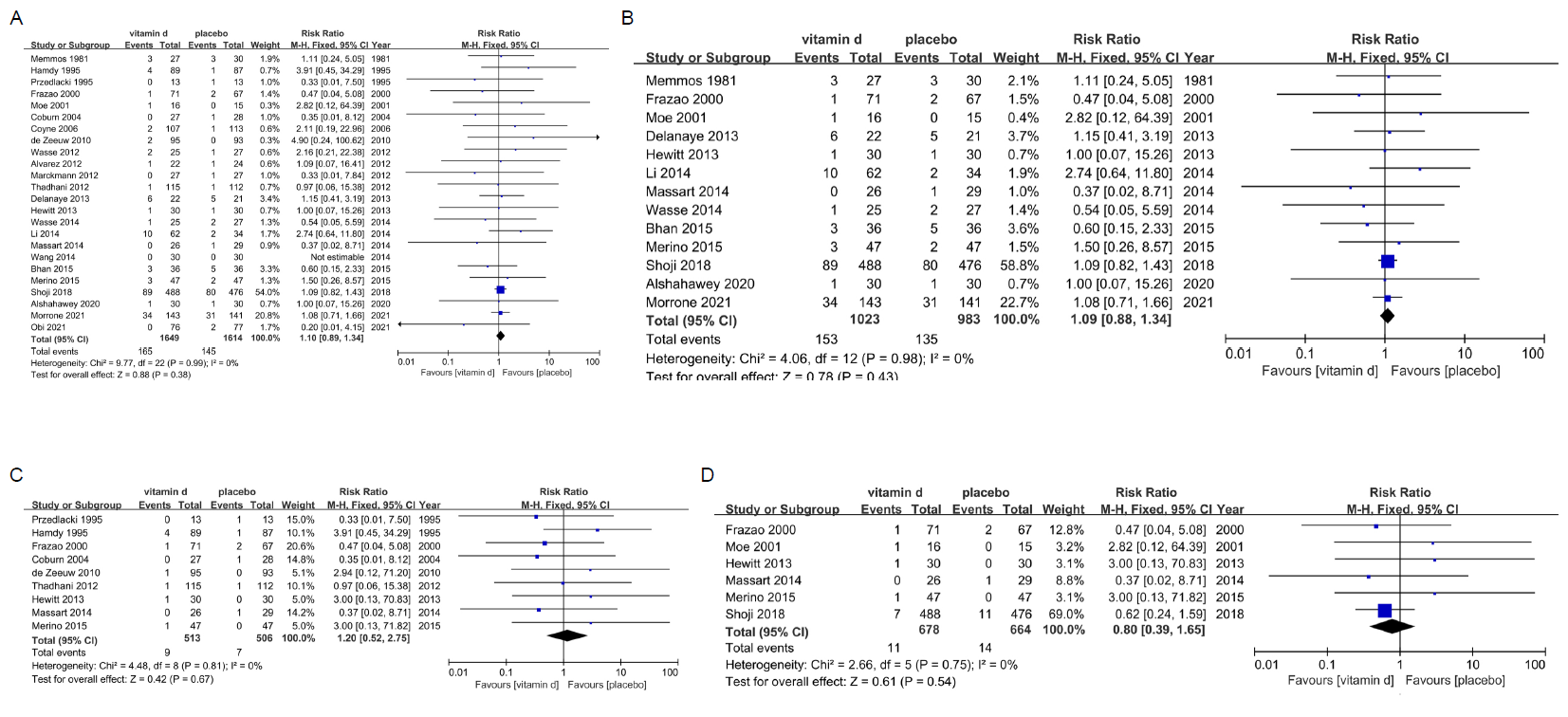


**Supplementary Figure 11.** Effects of vitamin D on all-cause mortality in CKD population. (A): Forest plot showing effects of vitamin D on all-cause mortality in all stages CKD population. (B): Subgroup analysis of all-cause mortality in dialysis population(late stage). (C): Forest plot showing effects of vitamin D on CVD mortality in all stages CKD population. (D): Subgroup analysis of CVD mortality in dialysis population(late stage).


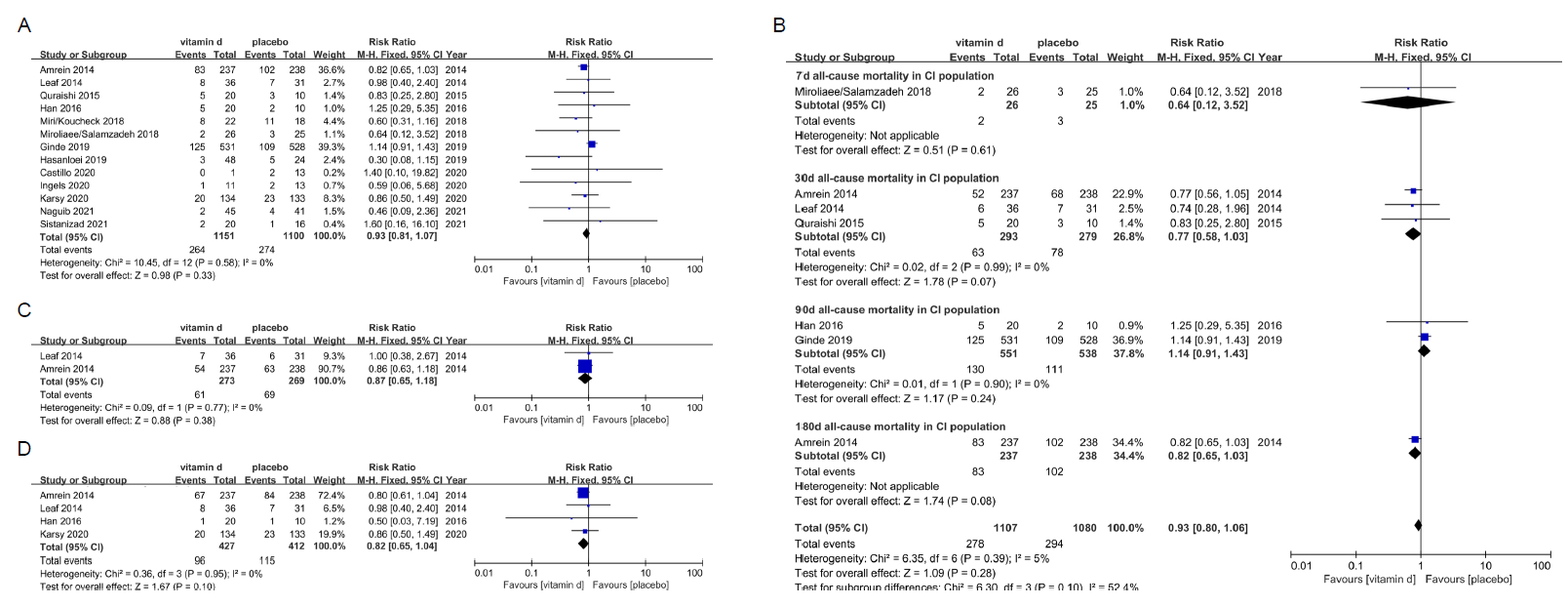


**Supplementary Figure 12.** Effects of vitamin D on all-cause mortality in critically ill population. (A): Forest plot showing effects of vitamin D on all-cause mortality in critically ill population. (B): Subgroup analysis of time-related all-cause mortality in critically ill population. (C): Subgroup analysis of ICU mortality in critically ill population. (D): Subgroup analysis of hospital mortality in critically ill population.


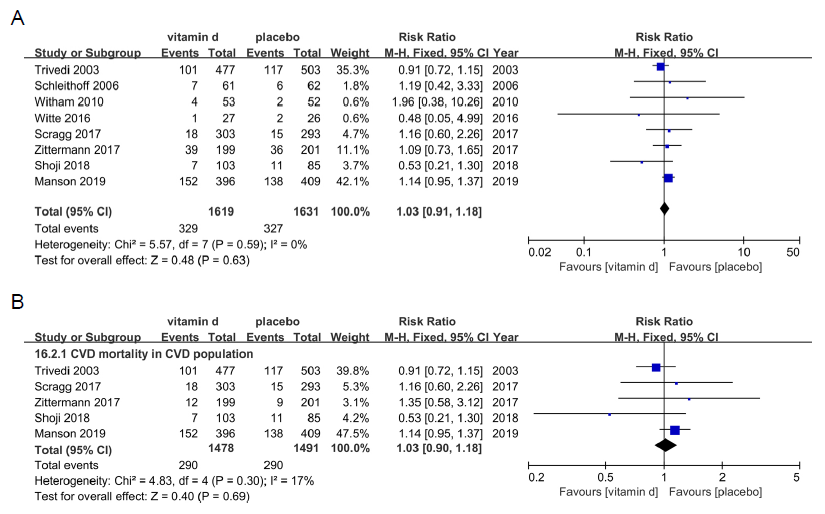


**Supplementary Figure 13.** Effects of vitamin D on mortality in CVD population. (A): Forest plot showing effects of vitamin D on all-cause mortality in CVD population. (B): Forest plot showing effects of vitamin D on CVD mortality in CVD population.


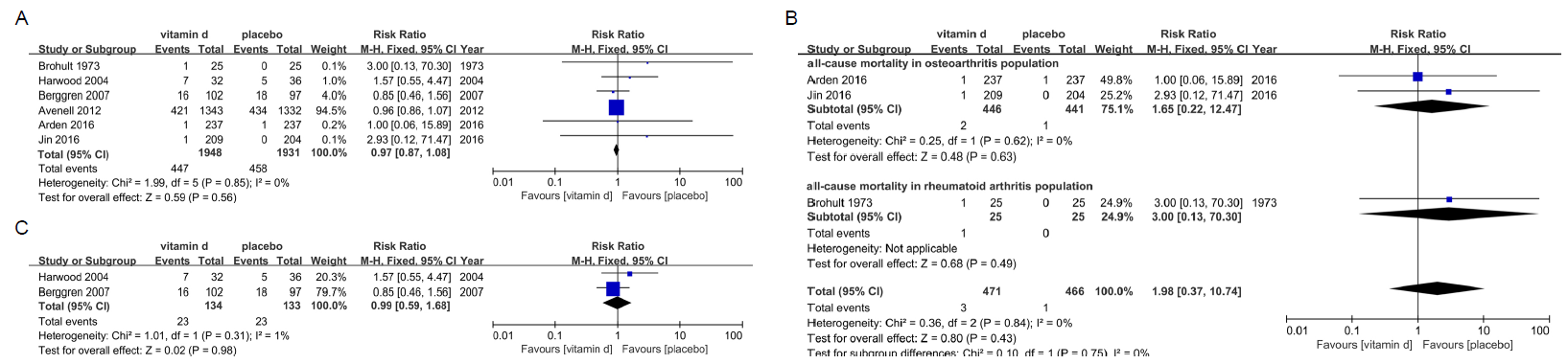


**Supplementary Figure 14.** Effects of vitamin D on all-cause mortality in musculoskeletal disease population. (A): Forest plot showing effects of vitamin D on all-cause mortality in all kinds of musculoskeletal disease population. (B): Subgroup analysis of all-cause mortality in arthritis population. (C): Subgroup analysis of all-cause mortality in population after fracture operation.


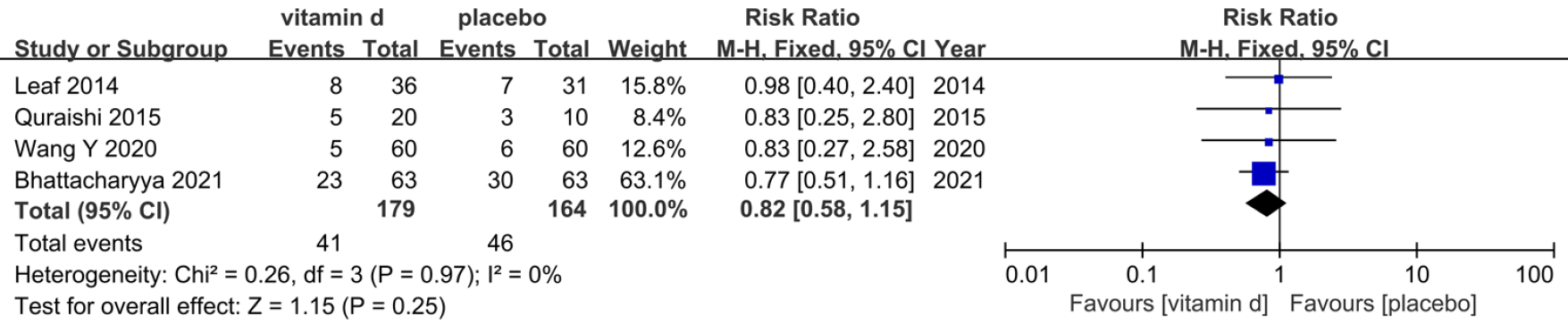


**Supplementary Figure 15.** Forest plot showing effects of vitamin D on all-cause mortality in sepsis population


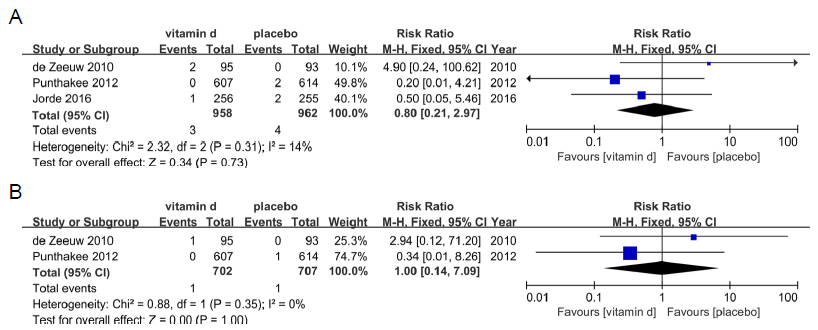


**Supplementary Figure 16.** Effects of vitamin D on mortality in T2DM population. (A): Forest plot showing effects of vitamin D on all-cause mortality in T2DM population. (B): Forest plot showing effects of vitamin D on CVD mortality in T2DM population.
